# Integrated mental health care and vocational rehabilitation to improve return to work rates for people on sick leave because of exhaustion disorder, adjustment disorder, and distress (the Danish IBBIS trial): study protocol for a randomized controlled trial

**DOI:** 10.1186/s13063-017-2273-0

**Published:** 2017-12-02

**Authors:** Rie Poulsen, Jonas Fisker, Andreas Hoff, Carsten Hjorthøj, Lene Falgaard Eplov

**Affiliations:** Mental Health Center Copenhagen, Mental Health Services Capital Region of Denmark, University of Copenhagen, Kildegårdsvej 28, 2900 Hellerup, Copenhagen Denmark

**Keywords:** Adjustment disorder, Exhaustion disorder, Distress, Return to work, Integrated services, Stress coaching, MBSR, Vocational rehabilitation, Prevention of recurrent sickness absence, RCT

## Abstract

**Background:**

Common mental disorders are important contributors to the global burden of disease and cause negative effects on both the individual and society. Stress-related disorders influence the individual’s workability and cause early retirement pensions in Denmark. There is no clear evidence that mental health care alone will provide sufficient support for vocational recovery for this group. Integrated vocational and health care services have shown good effects on return to work in other similar welfare contexts.

The purpose of the Danish IBBIS (Integreret Behandlings- og BeskæftigelsesIndsats til Sygemeldte) study is to examine the efficacy of (1) a stepped mental health care intervention with individual stress coaching and/or group-based MBSR and (2) an integrated stepped mental health care with individual stress coaching and/or group-based MBSR and vocational rehabilitation intervention for people on sick leave because of exhaustion disorder, adjustment disorder or distress in Denmark.

**Method/design:**

This three-armed, parallel-group, randomized superiority trial is set up to investigate the effectiveness of a stepped mental health care intervention and an integrated mental health care and vocational rehabilitation intervention for people on sick leave because of exhaustion disorder, adjustment disorder or distress in Denmark. The trial has an investigator-initiated multicenter design. Six hundred and three patients will be recruited from Danish vocational rehabilitation centers in four municipalities and randomly assigned into three groups: (1) IBBIS mental health care integrated with IBBIS vocational rehabilitation, (2) IBBIS mental health care and standard vocational rehabilitation, and (3) standard mental health care and standard vocational rehabilitation. The primary outcome is register-based return to work at 12 months. The secondary outcome measures are self-assessed level of depression (BDI), anxiety (BAI), distress symptoms (4DSQ), work- and social functioning (WSAS), and register-based recurrent sickness absence.

**Discussion:**

This study will contribute with knowledge on the consequence of the current organizational separation of health care interventions and vocational rehabilitation regarding the individual’s process of returning to work after sick leave because of exhaustion disorder, adjustment disorder or distress. If the effect on return to work, symptom level, and recurrent sick leave is different in the intervention groups, this study can contribute with new knowledge on shared care models and the potential for preventing deterioration in stress symptoms, prolonged sick leave, and recurrent sick leave.

**Trial registration:**

ClinicalTrials.gov, registration number: NCT02885519. Retrospectively registered on 15 August 2016). Participants have been included in the IBBIS trial for distress, adjustment disorder and exhaustion disorder since April 2016.

**Electronic supplementary material:**

The online version of this article (doi:10.1186/s13063-017-2273-0) contains supplementary material, which is available to authorized users.

## Background

Stress-related disorders, like exhaustion disorder, adjustment disorder and distress, are frequent causes of sick leave in Denmark and other high-income countries [1–3]. Stress-related disorders are associated with individual suffering, and people who are on sick leave due to stress-related disorders often leave work with feelings of shame, anger, poor self-esteem, and physical symptoms of stress [4]. Though distress and exhaustion disorder are not considered clinical diagnoses, they are often used as sick leave causes by general practitioners in Denmark and other countries [5], and distress is a known risk factor for development of mental disorders like depression and anxiety [6, 7]. Long-term sick leave because of mental problems is a heavy burden on society [8]. Psychiatric disorders have an estimated financial burden on the Danish economy at 3.4% of the Danish gross domestic product [9]. Common mental disorders, like depression, anxiety, and adjustment disorder, cause the largest financial burden because of their high prevalence [9, 10]. Around 20% of people on sick leave because of adjustment disorders have relapses of psychological disorders and reoccurring sick leave [11] and anxiety disorders are the most common reasons for early retirement in Denmark [12].

Adjustment disorders, exhaustion disorder, and distress can be characterized as significant emotional and behavioral problems in response to one or more identified stressors. These stressors can be work-related factors like poor leadership, insecure working conditions, bullying or conflicts. Employees with impaired work functioning nonetheless most often express that a combination of social, economic, and work-related factors have caused their mental problems [13].

Though work can impose difficult and stressful challenges, it also seems to be pivotal for people [14]. Manifest benefits from work (e.g., income) and latent benefits from employment (e.g., daily structure, social contact, professional identity, status, and activity) make work attractive for the individual [15], and this applies to people with mental health problems as well [16]. Furthermore, long-term sick leave and unemployment is a known stressor and a risk factor for poor mental health for the individual [17, 18]. Whereas short-term sick leave can be necessary for the stressed person, it is highly relevant to help individuals return to work and prevent long-term sickness absence and deterioration in mental health [19]. The aim of the IBBIS intervention is to improve sick leave beneficiaries’ process of returning to employment after long-term sick leave due to adjustment disorders, distress, and exhaustion disorder.

Few return to work interventions for people on sick leave because of stress-related disorders have proven effective. In 2016, Nigatu and colleagues concluded from a meta-analysis of diverse return to work interventions (e.g., problem-solving therapy, cognitive behavioral therapy (CBT), workplace-directed interventions) for people with common mental disorders that no significant difference was found in return to work rates for the overall intervention group compared with control. The meta-analysis showed a small and significant reduction in sick leave duration in the pooled intervention group, where participants returned to work 13 days earlier than controls. Interventions that have proven effective in single studies are problem-solving therapy interventions, multidisciplinary interventions and/or Mindfulness-based Stress Reduction (MBSR) interventions [20].

Arends and colleagues’ earlier Cochrane Review regarding interventions specifically for people with adjustment disorders concluded that CBT did not improve part-time or full return to work compared to treatment as usual. Whereas problem-solving therapy did improve distress according to the Four-Dimensional Symptom Questionnaire (4DSQ) [5] after 3 months and improved partial return to work, it did not improve full return to work [2]. A Danish study by Netterstrøm and colleagues have shown good effect on stress symptoms and return to work with a combined intervention of workplace-oriented stress coaching and MBSR courses [21]. This study had a relatively small sample size and was conducted without a prior sample size calculation. Despite the large effect sizes, there is a risk of random type I error, and the result should be replicated in a larger population.

Return to work following sick leave is a multifaceted and complex process [22]. Personal, structural, and work-related factors probably also play an important role in the trajectory of return to work [23, 24]. Thus, the process of recovering from mental health problems and the process of vocational recovery are intertwined, as the reintegration in the workplace affects the individual’s mental health just as well as mental health affects work reintegration [25]. Most return to work interventions can be characterized as psychological interventions addressing the resilience of individuals undergoing stressful circumstances with a cognitive approach [20]. Work-related problems are addressed indirectly through the worker. Novel evidence-based interventions for people with common mental disorders directly addressing workplace accommodations are scarce. The intervention Individual Placement and Support (IPS) is an American intervention with a strong emphasis on integration of treatment and vocational support. IPS has shown to be superior compared with standard services in people with severe mental illness attaining and maintaining work in a Swedish randomized controlled trial (RCT) [26, 27], but there is not yet solid evidence on how IPS can best be modified to suit a target group with common mental disorders. Recently, a large Norwegian study tested integrated employment support designed with an emphasis on IPS principles and a work-directed therapy (“At work and Coping, AWaC”). The study showed positive results regarding faster return to work for people with common mental disorders [28].

Rehabilitation after stress-related disorders seems to be an issue that lies in the borderland between the health care sector and the employment sector. In Denmark, vocational rehabilitation and work-directed interventions are governed by the local public office called the job center. The job centers handle the benefit case closely together with the provision of rehabilitation services [29]. The Organization for Economic Co-operation and Development (OECD) suggests that there is an unfortunate lack of coordination between the health care system and social insurance offices in Scandinavian countries [9]. The lack of coordination causes conflicting requirements and goals and a feeling of confusion and uncertainty for the individual on sick leave at a time where the individual lacks control and certainty [22, 30]. Intervention models that genuinely integrate services from the health care sectors and the employment sector in Scandinavia have not yet, to the authors’ knowledge, been tested on a population with stress-related disorders.

The aim of the IBBIS trial for exhaustion disorder, adjustment disorder, and distress is to test the effect on return to work from stepped mental health care and integrated stepped mental health care and vocational rehabilitation. By integrating and coordinating the two types of interventions, we aim to reduce counterproductive aims and services in both sectors, conveying a higher degree of collaboration about the participants’ mental and vocational recovery.

## Methods/design

### Aim

The aim of the randomized, three-armed, investigator-initiated, multicenter, parallel-group, superiority trial is to compare the effect on return to work of the following interventions: (1) IBBIS mental health care integrated with IBBIS vocational rehabilitation, (2) IBBIS mental health care and standard vocational rehabilitation or (3) standard mental health care and standard vocational rehabilitation. The primary hypothesis is that participants allocated to the IBBIS mental health care integrated with IBBIS vocational rehabilitation will have significantly faster return to work rates than people who are allocated to standard mental health care together with standard vocational rehabilitation. The secondary hypothesis is that IBBIS mental health care together with standard vocational rehabilitation will have a lesser but significant effect on return to work compared with standard mental health care together with standard vocational rehabilitation. The superiority of the IBBIS mental health care integrated with IBBIS vocational rehabilitation will also be tested by comparison with the IBBIS mental health care together with standard vocational rehabilitation intervention. The IBBIS vocational rehabilitation intervention alone will not be tested in this trial. We hypothesize that the superiority of the IBBIS interventions will be applicable for secondary outcomes and exploratory measures at 6-, 12-, and 24-month follow-ups so that (1) symptom level and presenteeism will be lower in participants allocated to IBBIS interventions and that (2) improvement in self-efficacy, quality of life, and functioning will be better in participants allocated to IBBIS interventions, and (3) satisfaction with services and number of weeks worked will be higher for participants allocated to IBBIS interventions.

The IBBIS trial is designed and reported in this article according to the Standard Protocol Items: Recommendations for Interventional Trials (SPIRIT) 2013 Statement (SPIRIT checklist and elaborated SPIRIT figure is provided as Additional file 1) [31], and the final results will be published according to the Consolidated Standards of Reporting Trials (CONSORT) criteria for Randomized Trials of Nonpharmacologic Treatment [32]. There is a parallel trial in the IBBIS project with identical research design for participants with depression and anxiety (reference to parallel IBBIS trial if possible).

### Setting

The interventions will be delivered by a cross-sector and multidisciplinary IBBIS team which is organized in collaboration between Mental Health Services in the Capital Region of Denmark and the following four municipalities: The City of Copenhagen, and the three suburban municipalities Gentofte, Gladsaxe, and Lyngby-Taarbæk. Participants are referred to the study by social security officers from job centers in the four municipalities, and the interventions are provided in locations other than the job centers and the mental health centers.

### Participants

Eligible participants in this trial are adults who are on sick leave from work or unemployment and have received sick leave benefit for at least 4 weeks or have started a sick leave benefit case which is estimated to last for at least 8 weeks. Participants must meet the criteria for exhaustion disorders according to the National Board of Health and Welfare in Sweden [33], Adjustment disorder according to the *International Classification of Mental and Behavioural Disorders, version 10* (ICD-10) [34] or distress according to the Four-Dimensional Symptom Questionnaire (4DSQ) [5]. Participants must also meet the following criteria to participate in the trial: be a resident of collaborating municipalities at baseline, be able to understand, speak and read Danish, be aged 18 years or older, and have given verbal and written consent to participate in the trial. Eligibility and determination of disorder are assessed by an IBBIS team member (nurse, physiotherapist, social worker, occupational therapist, psychologist or psychiatrist) who is trained to perform the assessment.

The IBBIS interventions are not designed to accommodate people in need of acute or highly specialized care. Thus, the potential participant will not be eligible if they meet the exclusion criteria: (1) the assessor determines the patients’ suicide risk to be high according to the MINI instrument [35] and the physician confirms this risk, (2) the patient meets the screening criteria for dementia according to The Mini-Mental State Examination (MMSE) screening instrument [36], (3) the patient abuses alcohol and/or other substances according to the assessor, (4) the patient has a severe, unstable, somatic condition (e.g., cancer, chronic obstructive pulmonary disease), (5) the patient needs secondary mental health care or (6) the patient is judged by job center staff to be at risk of displaying aggressive behavior. We wish to compare the IBBIS mental health care intervention alone with standard treatment. Thus, a potential participant will not be included in the study if they (7) do not accept to abstain from taking part in any psychotherapy or psychotherapy-like treatment outside the IBBIS intervention if they are allocated to IBBIS mental health care. Exclusion criteria 2–5 are only applied if the physician in the IBBIS team confirms the assessment and anticipates that the patient cannot benefit from the IBBIS interventions. In cases with high suicide risk, the participant will be referred to acute care services. People with alcohol and substance abuse problems will be offered referral to treatment if relevant.

### Recruitment, data collection, and data management

Recruitment takes place in two steps. First, case managers from the four job centers can refer Danish-speaking, adult citizens on sick leave from either work or unemployment to a psychiatric assessment if either the case manager, the citizen or the individuals’ general practitioner suspects a mental health condition to have caused the sick leave. The referral and assessment are voluntary. The results of the assessment will be shared with the individual’s general practitioner (GP) and the job center. The psychiatric assessment is based on three sources of information about the participant:

- Personal interview conducted by a care manager/psychologist and supervised by psychiatrist, guided by the following instruments:
  - ○ MINI International Neuropsychiatric Interview (MINI) [35]
  - ○ Standardized Assessment of Personality – Abbreviated Scale (SAPAS) [37]
  - ○ Attention deficit hyperactivity disorder (ADHD)-symptom checklist for adults (Adult Self-Report Scale, ASRS v1.1) [38]
  - ○ If dementia is suspected: Mini-Mental State Examination (MMSE) [36]
- Self-assessed symptoms: 4DSQ [5]
- Sick leave note issued by the GP

Assessors are IBBIS team members who are specially trained to use the above-mentioned instruments. Trial eligibility will be evaluated after the psychiatric assessment, and subsequently, assessment data will constitute baseline data at the time − 1 (see Table 1). The assessment process should not take more than 3 weeks but can be prolonged if one or more of the three types of information are missing.

**Table 1:** Standard Protocol Items: Recommendations for Interventional Trials (SPIRIT) figure: enrollment and data collection

	Baseline t_-1_	Randomization t_0_	6-month follow-up t_1_	12-month follow-up, t_2_	24-month follow-up t_3_
Informed consent	X				
Case Report Form (CRF) from personal interviews	X				
Randomization database		X			
Registration sheets	X	X	Continuous registration		
Self-assessment data	X		X	X	X
Register data		X	X	X	X

**Fig. 1:**
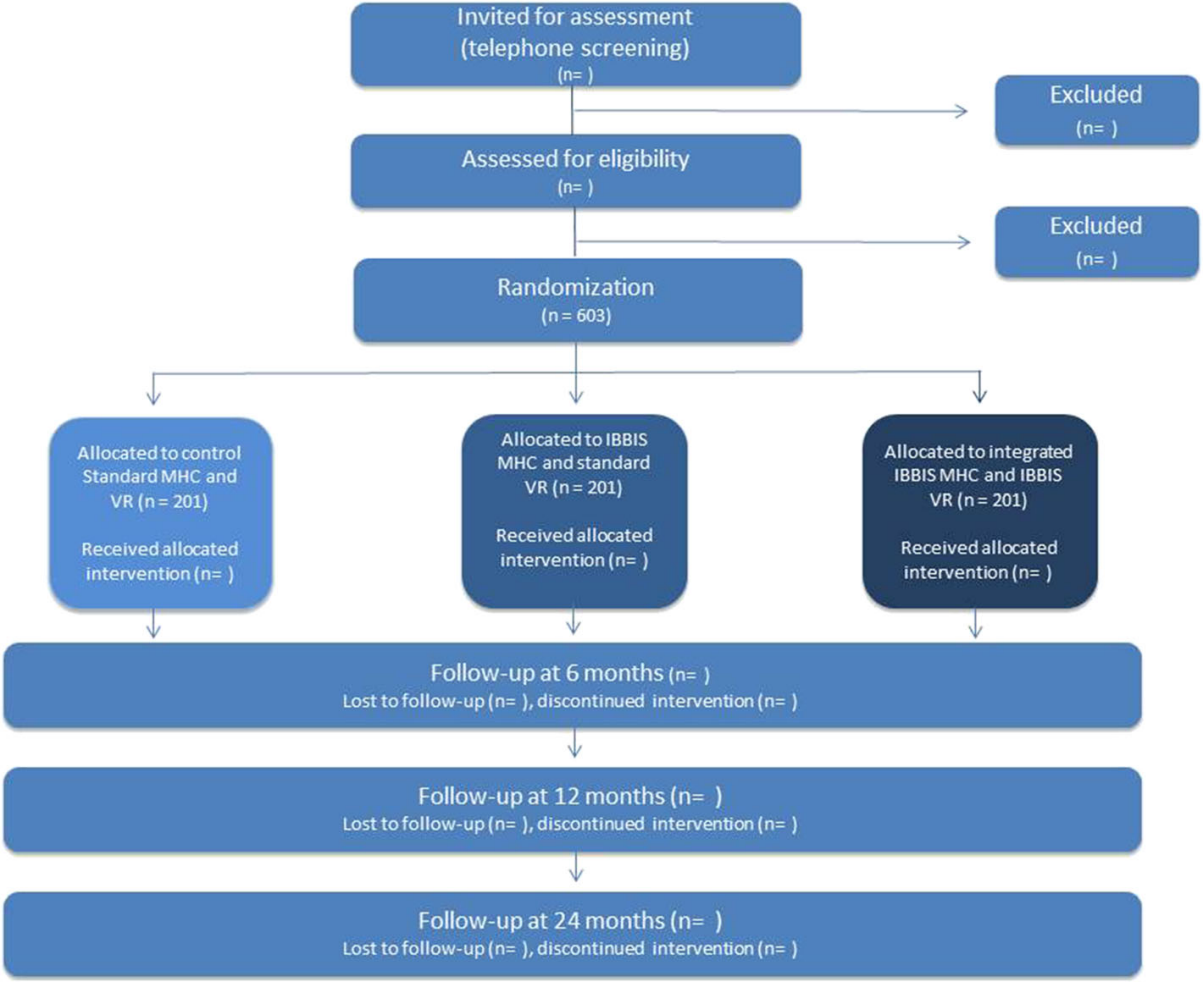
Flow chart for IBBIS participants

Second, individuals who meet the aforementioned trial criteria at assessment, and subsequently consent to participate, will be randomly allocated to an intervention by the assessor at t_0_. The results of the psychiatric assessment will be utilized in the treatment plan if the participant is allocated to treatment in IBBIS. Participants will be followed up at 6, 12, and 24 months after allocation (see Fig. 1 for the flowchart of participant timeline). Participants will be prompted to fulfill self-assessment questionnaires at each follow-up time through up to five personal contacts.

All electronic data (self-assessment, interview, and register data) are stored on secured servers at closed networks, and access to data is logged through unique login for an assigned list of IBBIS personnel. Physical data material (Case Report Forms with selected interview data) is stored in locked spaces, in locked facilities. Transfer of electronic data between staff members and other approved data managing institutions is carried out using only tunnel-encrypted e-mailing or encrypted UBS sticks.

### Randomization

Participants will be allocated to receive one of the following interventions: (1) IBBIS mental health care and standard vocational rehabilitation, (2) IBBIS mental health care integrated with IBBIS vocational rehabilitation or (3) standard mental health care and standard vocational rehabilitation. The allocation ratio between the three arms is 1:1:1. A centralized randomization will take place according to a web-based, computer-generated, allocation sequence with varying block sizes kept unknown to the assessors. Odense Patient data Explorative Network (OPEN) is responsible for the randomization, administrative personnel in the IBBIS team perform the online randomization, and the IBBIS team leader will assign the participant to interventions and professionals.

We expect that service delivery can vary from municipality to municipality and the process of gaining a new job from unemployment will take longer time than returning to an existing job. Previous research has shown that diagnosis is a possible predictor of return to work [39]. Thus, the randomization is stratified according to (1) municipality, (2) employment status at baseline (on sick leave from work vs. on sick leave from unemployment), and (3) diagnosis (adjustment disorder vs. distress vs. exhaustion disorder).

### Blinding

Due to the modalities of the IBBIS interventions, the participants and the professionals delivering the IBBIS interventions cannot be blinded to the group allocation. All outcomes are based on registries or self-assessed questionnaire data, and no assessor-based follow-up data will be obtained. Register data on employment status and income is created automatically through the national registries. Information on the participant’s sick leave benefit status is created through the job center management system, and benefits are granted and registered by the employment consultants in the IBBIS team.

Blinding of assessors is only relevant at the baseline interview, which takes place before group allocation. Referring personnel will likewise be blinded to the allocation sequence and block size to prevent them from anticipating the next group allocation. The researchers will be blinded to group allocation during the process of data analysis. Group allocation will be coded with names like X, Y, and Z to conceal the given intervention. The researcher will draw up conclusions at the 6- and 12-month follow-up based on the six scenarios where each group (X, Y or Z) has received IBBIS mental health care and standard vocational rehabilitation, integrated IBBIS mental health care and IBBIS vocational rehabilitation, and standard mental health care and vocational rehabilitation. After this, the blinding will be broken. The researcher performing analysis at 24-month follow-up will not be blind to group allocation, as any possible differences between groups will be revealed after 12-month follow-up.

### Interventions and comparisons

The IBBIS intervention team is constituted by (1) three and a half full-time care managers who are nurses, occupational therapists, physiotherapists or social workers with mental health care experience and a minimum of 1 year certified training in CBT, (2) three and a half full-time employment consultants who are social insurance officers from the job centers, and (3) equivalent to 0.75 full-time psychiatrists (alternatively, general practitioner or psychologist). Care managers, psychiatrists, and psychologists are employed in the Mental Health Services, and employment consultants are employed in the job centers of the four municipalities. Care managers have a maximum momentary caseload of 25, and employment consultants have a maximum momentary caseload of 20. The IBBIS mental health care intervention is expected to have an average duration of 4 months, and the duration of the IBBIS vocational rehabilitation is expected to average 7 months.

The team delivers two separate interventions (1) IBBIS mental health care alone and (2) IBBIS mental health care integrated with IBBIS vocational rehabilitation. Both interventions are carried out with a great emphasis on participant involvement, shared decision-making, and involvement of the participants’ relatives. A fidelity scale (unpublished, available through the corresponding author) is developed and used for biannual fidelity reviews to ensure program adherence and continuous focus on program implementation and improvement. The fidelity reviews are based on observations and individual and group interviews of professionals, management, and participants, as well as a review of 10 participant cases. Once program fidelity is achieved future fidelity reviews will be conducted annually.

### IBBIS mental health care and standard vocational rehabilitation

IBBIS mental health care is delivered as manualized stepped care. The participant will be offered treatment options according to a stepped care plan, offering the least invasive and least resource-demanding effective treatment first. Intervention modalities will be offered according to the stepped care plan, see Table 2. One or more of the following treatment options will be provided:

**Table 2:** Stepped care algorithm for adjustment disorder, exhaustion disorder, and distress

Step	Disorder	Treatment
1	Mild stress disorder (4DSQ distress-subscale 10–20) for < 2 months or Moderate stress disorder (4DSQ distress-subscale > 20) for < 1 months or Adjustment disorder < 2 months	Bibliotherapy Individual psychoeducation Monitoring by care manager Involvement of relatives
2	Moderate stress disorder (4DSQ distress-subscale > 20) for > 1 month or Mild stress disorder (4DSQ distress-subscale 10–20) for > 2 months or Adjustment disorder for > 2 months and 4DSQ 10–20 distress-subscale or Adjustment disorder for > 1 month and 4DSQ distress-subscale > 20	Stress coaching Bibliotherapy Individual psychoeducation Monitoring by care manager Involvement of relatives
3	Exhaustion disorder according to the National Board of Health and Welfare in Sweden	MBSR Stress coaching Bibliotherapy Individual psychoeducation Monitoring by care manager Involvement of relatives

- Care plan produced in collaboration with the care manager and participant and in accordance with treatment guidelines for the symptom severity and relevant step (step 1–3)
- Regular monitoring by the care manager of progression in symptom level (according to 4DSQ) minimum every fortnight to ensure timely changes in the treatment plan and step if participants deteriorate [5] (steps 1–3)
- Individual psychoeducation with a self-management approach by the care manager. The psychoeducation aims at providing general information about symptoms and coping strategies to normalize and provide help for self-help (steps 1–3)
- Bibliotherapy: supplementary psychoeducational disease-specific written material which aims at providing general information about symptoms and coping strategies to normalize and provide help for self-help (steps 1–3)
- Involvement of relatives by the care manager (steps 1–3)
- Stress coaching conducted by the care manager inspired by the intervention in Netterstrøm (2013) [21]. The stress coaching intervention is a structured, seven-session individual intervention with focus on immediate stress reduction, identification of stressors, changing coping strategies and restoring balance (step 2)
- An eight-session, group-based, Mindfulness-based Stress Reduction (MBSR) program [40] conducted by certified MBSR teachers (step 3)

The psychiatrist and/or the psychologist of the IBBIS team are responsible for:

- Supervision of care managers
- Initiation of non-medical treatment (can be delegated to the care manager under supervision)
- Collaboration with the participant’s GP and other possible treatment providers

Participants will receive standard vocational rehabilitation services from the local job center along with continuous control of the grounds for receiving sick leave benefit. The IBBIS team will not collaborate with job center personnel.

### Integrated IBBIS mental health care and IBBIS vocational rehabilitation

The mental health care in intervention in the integrated intervention is identical with that which is described in the IBBIS mental health care above. The concurrent vocational rehabilitation in IBBIS is composed of the following elements, which are delivered to meet the participant’s individual needs for vocational recovery:

- Vocational assessment of the participant’s work capacity and barriers in relation to work with focus on readiness for return to work [41], work role functioning [42], and return to work self-efficacy [43]
- Vocational rehabilitation plan produced in collaboration with the participant and in compliance with the vocational rehabilitation manual
- Problem-solving support in returning to a current workplace and preventing recurring sick leave inspired by Dutch guidelines and the intervention *SHARP-at work*. The support is focused on quick, stepwise return to work and problem-solving of issues related to the work-place which are barriers for return to work or impose risk factors for recurring sick-leave [44, 45]
- Job-search support with a focus on the best possible job match inspired by Individual Placement and Support (IPS) in accordance with the slightly moderated IPS principles (1) focus on competitive employment, (2) integration of mental health and employment services, (3) strong attention to participant preferences, (4) counseling about benefit programs and supported work accommodations, (5) rapid job search, (6) systematic job development, and (7) time-unlimited support for work retention [46]
- Case management according to Danish sick leave benefit legislation with continuous assessment of the justification of the type and duration of sick leave benefit
- Coordination, where relevant, with other public authorities who provide social services
- Involvement of relatives

Consistency between goals in treatment and vocational rehabilitation is crucial [22, 30]. Several integrational elements ensure coherence in the participants’ process of returning to work and recovering from mental problems in the integrated IBBIS intervention:

- At least one meeting between the participant, the employment consultant, and the care manager where a joint plan for return to employment and the support from the IBBIS team is decided upon
- Co-location of all team members
- Multidisciplinary supervision of care managers and employment consultants together to enhance a continuous focus on the shared goals of the participants

The integrated services are based on the theoretical framework *relational coordination* by Jody Gittell in which timely and problem-solving communication between different professionals is created by focusing on shared goals, shared knowledge and mutual respect [47]. Unfortunately, it has not been possible to establish a shared electronic folder for IBBIS staff from different organizations to use due to separate secure IT systems and, hence, written communication across sectors and municipalities can only be shared through encrypted e-mails according to national guidelines to conform to the Act on the processing of Personal Data.

### Training and supervision

Employment consultants and care managers have all attended a 4-week training course in April 2016; 1 week of joint training and 3 weeks of training in their monodisciplinary groups. Care managers are trained in all aspects of the IBBIS mental health care intervention with a special focus on psychiatric assessments and CBT. Likewise, employment consultants are trained in all aspects of the IBBIS vocational rehabilitation intervention with a special focus on the problem-solving method and job development. Care managers have weekly, case-based supervision and stress coaching supervision every fortnight, the employment consultants have weekly supervision, and the team has monthly, case-based, cross-disciplinary supervision.

### Standard mental health care and standard vocational rehabilitation

Participants who will be allocated to the control group will receive standard health care by their GP and standard services in the job center. Adjustment disorder, exhaustion disorder, and distress are commonly used as reasons for sick leave by the GP, but are nonetheless not disorders that the public health care system is obliged to provide treatment for. A large number of private companies offer treatment of stress-related disorders in Denmark through an emerging market of insurance companies and out-of-pocket offers. These have been characterized as very diverse and lacking an evidence base [48].

GPs can, with supervision, offer up to seven therapy sessions to patients with social or psychological complaints. It is estimated that 89% of Danish GPs offer therapy to their patients and 49% of the GPs patients receiving therapy are registered with stress or adjustment disorder. The therapy is often very short term, as 36% of patients only receive one session and 73% receive three or fewer therapy session [49]. Local health authorities are not obliged to offer help to people who suffer from stress-related disorders, but two out of three municipalities in Denmark offer self-management courses [50] or stress management groups to people who can self-refer with mental problems.

The job centers offer a variety of courses and support and manage the sick leave benefit case according to government legislation, which requires regular follow-up every 4 weeks, reassessment of the sick leave diagnosis after 22 weeks, self-management courses and support to gradual return to work (in paid or unpaid jobs).

Collaboration between the job center and the health care system is minimal and conducted through standardized sick leave certificates from the GP to the job center. Representatives from the health care system (other than the individuals’ own health care providers) can be used in reassessment of the individuals’ sick leave case.

### Outcomes

The primary outcome is time from baseline to the event *return to work,* within 12 months after baseline. Work is defined as having four consecutive weeks of working with a salary and with no concurrent vocational benefits. Benefit and income status is retrieved from the Danish DREAM database and the electronic income register [51]. The DREAM database is administered by Danish Agency for Labour Market and Recruitment and can be linked to a range of different registers, including the Danish Income Register. Returning to or achieving a flex-job, a type of subsidized work, is also defined as returning to work for participants who are entitled to flex-job when they enter the trial. The work-related, symptom-based and functional secondary outcomes are presented in Table 3. All explorative and safety measures are presented in Table 4.

**Table 3:** Primary and secondary outcomes and data collection

	Data source	Outcome	Baseline	6-month follow-up	12-month follow-up	24-month follow-up
Primary	DREAM database	Time from baseline to RTW			X	
Secondary	DREAM database	Proportion in ordinary work			X	
	DREAM database	Time from baseline to RTW		X		X
	DREAM database	Time from the first day of RTW until possible recurrent sick leave				X
	Questionnaire	Difference in depressive symptoms measured by Beck Depression Inventory (BDI-II) [52]	X	X		
	Questionnaire	Difference in anxiety symptoms measured by Beck Anxiety Inventory (BAI) [55]	X	X		
	Questionnaire	Difference in stress symptoms measured by Cohen Perceived Stress 10-item Scale (PSS) [77]	X	X		
	Questionnaire	Social and work-related function measured by WSAS [57]	X	X		

*RTW* return to work, *WSAS* Work and Social Adjustment Scale

**Table 4:** Explorative outcomes and safety measures

Data source	Outcome	Baseline	Follow-up		
			6 months	12 months	24 months
DREAM database	Weeks of work from baseline to current follow-up			X	X
Questionnaires	Symptoms of distress, anxiety, depression, and somatization by Four-Dimensional Symptom Questionnaire (4DSQ) [5]	X	X	X	X
	Depressive symptoms measured by Beck Depression Inventory-II (BDI-II) [52]	X		X	X
	Anxiety symptoms measured by Beck Anxiety Inventory (BAI) [55]	X		X	X
	Stress-symptoms measured by Cohen Perceived Stress 10-item Scale (PSS) [77]	X		X	X
	Social and work-related function measured by WSAS [57]	X		X	X
	Burn-out symptoms measured by Karolinska Exhaustion Scale (KES) [58]	X	X	X	X
	Health-related quality of life measured by EQ-5D-5L [78]	X	X	X	X
	General quality of life measured by Flanagan’s QOLS [61]	X	X	X	X
	Self-efficacy concerning symptoms measured by IPQ subscale on personal control [62]	X	X	X	X
	Return to work self-efficacy measured by RTW-SE [63]	X	X	X	X
	General self-efficacy measured by General Self-efficacy Scale (GSS) [64]	X	X	X	X
	Client satisfaction with treatment measure measured by CSQ-8 [65]		X		
	Presenteeism measured by Stanford Presenteeism Scale (SPS) [79]		X	X	X
	Use of therapy and therapy-like services outside IBBIS		X	X	X

*CSQ-8* Client Satisfaction Questionnaire, *EQ-5D-5L* European Quality of Life Five Dimension Five Level version, *IPQ* Illness Perception Questionnaire-Revised, QOLS Quality of Life Scale, *RTW-SE* Return to Work Self-efficacy

The Beck Depression Inventory (BDI–II) consists of 21 items to assess the intensity of depression in clinical and normal patients. Each item is a list of four statements (0 to 3) arranged in increasing severity about a particular symptom of depression [52]. The Beck Anxiety Inventory (BAI) is a 21-item general questionnaire for anxiety, measuring symptoms during the last week rated on a four-point Likert-scale from 0 to 3 [53]. The BDI and BAI has shown excellent psychometric properties, with internal consistency around 0.9 [54, 55]. Cohen’s Perceived Stress Scale (PSS) is a global measure of perceived stress. The scale was originally a 14-item questionnaire, and it has later been moderated to a 10-item questionnaire which shows improved and satisfactory psychometric properties [56]. The Work and Social Adjustment Scale (WSAS) is a simple, reliable, five-item scale which measures functional impairment related to an identified problem [57], which is defined in this trial as “psychological symptoms.”

The Four-Dimensional Symptom Questionnaire (4DSQ) is a 50-item questionnaire designed to assess common psychological symptoms in the last week and has a special focus on distinguishing general distress from depression, anxiety, and somatization [5]. The Karolinska Exhaustion Scale (KES) 26-item version measures the degree of exhaustion disorder and the four inter-related dimensions of exhaustion disorder according to the Swedish National Board of Health and Welfare: lack of recovery, cognitive exhaustion, somatic symptoms, and emotional distress [58, 59]. The European Quality of Life Five Dimension Five Level version (EQ-5D-5L) is a measure of health status in five domains: mobility, self-care, usual activities, pain/discomfort and anxiety/depression and also includes a Visual Analogue Scale from 0 (worst imaginable health status) to 100 (best imaginable health status) [60]. Flanagan’s QOLS is a 16-item instrument that measures five conceptual domains of quality of life: material and physical wellbeing, relationships with other people, social, community and civic activities, personal development and fulfillment, recreation, and independence [61]. The six-item Personal Control subscale from the revised version of the Illness Perception Questionnaire (IPQ-R) is used to evaluate the participant’s self-efficacy regarding symptom management [62]. Return to work self-efficacy (RTW-SE) is an 11-item measure for self-efficacy beliefs regarding return to work where respondents are asked to respond to statements about their jobs, imagining that they would start working tomorrow in their present emotional state [63]. The General Self-Efficacy Scale is a 10-item psychometric scale that is designed to assess optimistic self-beliefs to cope with a variety of difficult demands in life [64]. The Client Satisfaction Questionnaire (CSQ-8) is an eight-item questionnaire which is used to measure the participants’ satisfaction with mental health care services and vocational rehabilitation [65]. Presenteeism refers to the state where a person attends work while being sick [66] and is used as a proxy measure for returning to work while having reduced workability.

### Sample size and power calculation

The sample size of this trial is based on the primary outcome return to work rate (hazard ratio (HR)). There are to the authors’ knowledge no comparable Danish studies, and the sample size estimates are based on Dutch studies of comparable interventions for populations on sick leave with common mental disorders. The desired type II error risk is set at 10% (power = 90%). The mean number of days from baseline to return to work in the control group is conservatively estimated to be 210 days [67–69]. Due to multiple testing, as we will make comparisons between all three study arms, we Bonferroni correct the type I error risk (*α*) to 0.0167. An HR of 1.5 is estimated to be clinically relevant [70–72], and participants will be recruited through 639 days and followed for 365 additional days. With an allocation ratio of 1:1:1 we need 201 participants in each of the three arms to reject the null hypothesis that the return to work rate is equal in the control group, the IBBIS mental health care intervention, and the integrated IBBIS mental health care and IBBIS vocational rehabilitation intervention, respectively. If we fail to include 603 participants, the statistical power can be lowered to 80% and thus only 468 participants will be needed.

Power calculations (Tables 5 and 6) indicate that a sample size of 201 participants per group will be adequate to detect relevant significant differences in the secondary outcome measures with minimum 80% power.

**Table 5:** Power calculation for binary secondary outcomes

Outcome	Expected proportion in control group	Clinically relevant proportion in intervention group	*α*	Power	Test	Reference
Proportion achieving > 4 four weeks of ordinary job	0.65	0.80	0.0167	0.838	χ^2^ test	[67–69, 80]
Proportion of > 4 weeks recurrent sick leave absence among participants who returned to work	0.19	0.08	0.0167	0.801	χ^2^ test	[11]

**Table 6:** Power calculation for linear secondary outcomes

Outcome	*δ* clinically relevant difference in mean	*σ* expected standard deviation	*α*	Power	Test	Reference
Difference in depressive symptoms measured by Beck Depression Inventory (BDI)	4	11	0.0167	0.893	*t* test	[81–86]
Difference in anxiety symptoms measured by Beck Anxiety Inventory (BAI)	4	12	0.0167	0.826	*t* test	
Difference in stress symptoms measured by Cohen’s Perceived Stress Scale (PSS)	5	8	0.0167	1.000	*t* test	[87–89]
Social- and work-related function measured by WSAS	4	10	0.0167	0.946	*t* test	[90]

*WSAS* Work and Social Adjustment Scale

All sample size and power calculations are conducted in the PS: Power and Sample Size Calculation software [73].

### Statistical analysis plan

The primary objective of this superiority trial is to test if there is any difference in time from baseline to the event *return to work* between the three groups at 12-month follow-up time; the null hypothesis being that there is no difference. Because the primary outcome data is collected as register data, the data is expected to be complete. Kaplan-Meier survival curves will be computed, and the differences between the three intervention groups will be tested with a Cox proportional hazards regression analysis to estimate the treatment effect as HR with 95% confidence intervals. Cox regression analysis will also be used for the secondary outcomes at 24-month follow-up: “time from return to work to recurrent sick leave” for the subpopulation of individuals who have started working and “time from baseline to return to work.”

The continuous secondary outcomes at 6-month follow-up BDI, BAI, PSS, and WSAS are used with a repeated measurement design and the difference in the individuals’ scores between measurements will be analyzed by using linear mixed models with repeated measures and unstructured covariance matrix if possible. All participants will be included in the analysis according to the intention-to-treat principle. Missing data from the questionnaire-based instruments will be imputed with multiple imputations if we can assume that data are missing at random or missing completely at random*.* The effect of missing data will, furthermore, be assessed by sensitivity analyses. All statistical tests are two-sided. All exploratory continuous outcomes will be analyzed by the same method.

A non-parametric model will be used in situations where the scores are not normally distributed. The binary outcome *proportion in ordinary work* will be analyzed with logistic regression. All models will be adjusted for the stratification variables. We will assess the potential interaction between time and intervention for continuous secondary outcomes.

## Discussion

This paper describes the study protocol of a randomized controlled trial comparing (1) IBBIS mental health care integrated with IBBIS vocational rehabilitation, (2) IBBIS mental health care and standard vocational rehabilitation, and (3) standard mental health care and standard vocational rehabilitation for people on sick leave because of distress, adjustment disorder or exhaustion disorder. Stress-related conditions are frequent causes of sick leave in Denmark with great costs for the individual and society. This trial will test two new targeted approaches to mental health care and vocational rehabilitation and the integration of these interventions to reduce the burden of these conditions.

This randomized controlled trial is designed with great emphasis on minimizing bias and reporting is done in accordance with SPIRIT guidelines [31]. The large sample size and, hence, high statistical power allows for detection of relevant differences in both primary and secondary outcomes. The randomization is in accordance with high methodological standards. There are nonetheless some methodological challenges.

Firstly, we expect that some contamination between the IBBIS mental health care intervention and the integrated IBBIS mental health care and IBBIS vocational rehabilitation intervention will occur because care managers might be inclined to provide IBBIS mental health care with an undesirable emphasis on vocational recovery because of their close collaboration with employment consultants regarding the participants in the integrated intervention. To minimize the risk of contamination for participants in the IBBIS mental health care and standard vocational rehabilitation intervention care managers are prompted to avoid collaboration with regular job center case managers about individual cases. Secondly, participants and professionals are not blinded to group allocation, and there is also a risk of both performance bias and subject-expectancy bias. These likely biases are difficult to prevent and will be included in the interpretation of the results.

Thirdly, implementation of structured interventions in multicenter designs have previously shown to be difficult; several context-factors affect the implementation of the intervention [74], and some variation in the delivered services between the Danish municipalities is expected [75]. We attempt to minimize the bias from the possibly skewed implementation by stratifying the randomization for municipality. To address the possible differences in effects between municipalities, we will, furthermore, conduct fidelity reviews to explicate differences in implementation.

Fourthly, multidisciplinary teams have previously shown difficult to establish [75], and the collaboration in integrated care can be difficult to implement as it has to be established and maintained at the macro, meso, and micro level in all municipalities [76]. Thus, we expect the teams to perform better at the end of the trial period than at the beginning, which can explain a missing or minimal effect from the interventions. This will be examined by analyzing the possible interaction between intervention and time.

Fifthly, standard mental health care and standard vocation rehabilitation for people with stress-related disorders are very scarcely described in Denmark. Thus, a limitation in the study design is the limited knowledge about the quality and quantity of the control intervention. To improve the possibilities for comparison between the three interventions three questions about the participants’ use of therapy and therapy-like services outside IBBIS have been added to the self-assessment scheme.

Sixthly, a limitation in the study design is the fact that we cannot measure the effect of the IBBIS vocational rehabilitation alone. Unfortunately, a four-armed design was not feasible regarding economy and sample size, and a 2 × 2 factor design is not suitable when the intervention components are expected to interact in synergy in the integrated intervention. We prioritize to test the efficacy of the IBBIS mental health care intervention as we expected improved treatment to be a necessity for improved return to work.

If this trial shows that the IBBIS mental health care intervention is superior to standard treatment, these positive results will support the further development of enhanced community-based mental health care for people on sick leave, and a wider implementation of treatment teams similar to IBBIS can be recommended. If this trial shows that integrated IBBIS mental health care and IBBIS vocational rehabilitation is superior to standard treatment or IBBIS mental health care alone, the positive results will support the assumption that integrated care is not only a perceived need from the target group, but also an effective way of supporting people in their vocational recovery. If the standard intervention is superior regarding return to work, we have further incentive to attempt to improve treatment and vocational care; it can be considered if return to work rates has reached a maximum.

This study can contribute with new knowledge on integrated vocational and health care interventions in welfare societies with separate health care and occupational sectors, and prevention of recurrent sickness absence among people with distress, adjustment disorder or exhaustion disorder.

### Trial status

The IBBIS trial for distress, adjustment disorder, and exhaustion disorder was initiated in April 2016, and as of November 7, 2017, 426 participants have been recruited. This protocol is in version 2.0. Trial recruitment is expected to end on 31 December 2017.

## Additional file

Additional file 1: SPIRIT 2013 Checklist: recommended items to address in a clinical trial protocol and related documents^*^. (DOCX 44 kb)

## Abbreviations

4DSQ: Four-Dimensional Symptom Questionnaire
BAI: Beck Anxiety Inventory
BDI-II: Beck Depression Inventory II
CSQ-8: Client Satisfaction Questionnaire
DREAM: Danish Register for Evaluation of Marginalization
EQ-5D-5L: European Quality of Life Five Dimension, Five level version
GP: General practitioner
GSS: General Self-efficacy Scale
IPQ-R: Illness Perception Questionnaire-Revised
IPS: Individual Placement and Support
KES: Karolinska Exhaustion Scale
MINI: Mini International Neuropsychiatric Interview
OPEN: Odense Patient data Explorative Network
PSS: Cohen’s Perceived Stress Scale
QOLS: Quality of Life Scale
RTW-SE: Return to Work Self-efficacy
SAPAS: Standardized Assessment of Personality: Abbreviated Scale
SPS: Stanford Presenteeism Scale
WSAS: Work and Social Adjustment Scale
